# Novel approaches in the management of pancreatic ductal adenocarcinoma: potential promises for the future

**DOI:** 10.1186/s13045-015-0141-5

**Published:** 2015-05-03

**Authors:** Gaurav Goel, Weijing Sun

**Affiliations:** Division of Hematology-Oncology, Department of Medicine, University of Pittsburgh Cancer Institute, University of Pittsburgh School of Medicine, 5150 Centre Avenue, Fifth Floor, Pittsburgh, PA 15232 USA

**Keywords:** Pancreatic ductal adenocarcinoma, Advanced pancreatic cancer, Metastatic pancreatic cancer, Locally advanced pancreatic cancer, Novel therapies, Biomarkers

## Abstract

Despite a few breakthroughs in therapy for advanced disease in the recent years, pancreatic ductal adenocarcinoma continues to remain one of the most challenging human malignancies to treat. The overall prognosis for the majority of patients with pancreatic cancer is rather dismal, and therefore, more effective treatment options are being desperately sought. The practical goals of management are to improve the cure rates for patients with resectable disease, achieve a higher conversion rate of locally advanced tumor into potentially resectable disease, and finally, prolong the overall survival for those who develop metastatic disease. Our understanding of the complex genetic alterations, the implicated molecular pathways, and the role of desmoplastic stroma in pancreatic cancer tumorigenesis has increased several folds in the recent years. This has facilitated the development of novel therapeutic strategies against pancreatic cancer, some of which are currently under evaluation in ongoing preclinical and clinical studies. This review will summarize the existing treatment approaches for this devastating disease and also discuss the promising therapeutic approaches that are currently in different stages of clinical development.

## Introduction

Pancreatic ductal adenocarcinoma (PDAC) is the fourth leading cause of cancer-related mortality in the US for both men and women [1]. In 2015 alone, it is estimated that 48,960 new cases of pancreatic cancer will be diagnosed in the US and 40,560 patients will die of this disease. As such, PDAC remains one of the most challenging malignancies with a dismal prognosis and limited therapeutic options. The 5-year survival rate for pancreatic cancer (all stages combined) is around 7%, which is the lowest among all different cancer sites. At the time of initial pancreatic cancer diagnosis, approximately 9% of patients present with localized disease, 28% have regional spread, and the remaining 53% of patients already have distant spread of their disease. There has been a very limited clinically meaningful improvement in survival rates for this disease during the past two decades. The poor prognosis of PDAC is largely attributed to delayed diagnosis due to nonspecific symptoms in the early stages of the disease, biological aggressiveness leading to rapid metastases, lack of effective screening methods, and resistance to radiation and chemotherapies.

It is now well established that PDAC is driven by alteration of multiple genes that regulate pathways and processes in the tumor cells and the neighboring microenvironment [2]. Despite our improved understanding of the molecular events underlying the multistep carcinogenesis of PDAC, the progress made towards improving the survival rates of these patients has been extremely slow. At present, multiple novel treatment strategies targeted against PDAC are under preclinical and clinical evaluation. This review will summarize the existing treatment approaches for this devastating disease and also discuss the promising therapeutic strategies that are currently in the different stages of clinical development.

### Risk factors

The known risk factors that increase the likelihood of developing PDAC include cigarette smoking [3], alcohol abuse [4], high fat diet [5], and certain trace elements [6]. It is estimated that cigarette smoking doubles the risk of developing PDAC and accounts for approximately 20%–25% of the cases [3]. Chronic pancreatitis is also associated with an increased risk of PDAC, especially among smokers [7]. It has also been noted that the majority of patients with PDAC develop diabetes mellitus which is usually diagnosed in the preceding 1–2 years or concomitant with the new cancer diagnosis [7]. It is not entirely clear whether diabetes is a predisposing factor or a manifestation of PDAC itself. Obesity, which predisposes to insulin resistance, might be a common link between the two.

Approximately 5%–10% of patients with PDAC report a history of pancreatic cancer diagnosis in their family member [8]. The genetic syndromes such as familial breast cancer (*BRCA2*, *BRCA1*, and *PALB2*), the Peutz-Jeghers syndrome (*LKB1*/*STK11*), the familial atypical multiple mole melanoma (FAMMM) syndrome (p16/*CDKN2A*), hereditary pancreatitis (*PRSS1*), and the lynch syndrome (*MLH1*, *MSH2*, *MSH6*, *PMS2*) are also associated with an increased risk of developing PDAC [8,9]. Thus, patients with a family history of pancreatic cancer or these mutation carriers should undergo appropriate screening, as per the guidelines provided by the International Cancer of the Pancreas Screening Consortium [10].

### Genetics and molecular pathogenesis

Pancreatic cancer most commonly originates in the exocrine cells of the pancreas [11]. Among the exocrine tumors, ‘ductal adenocarcinoma’ is the most frequently encountered pathological subtype and accounts for more than 90% of the cases. Initiation and development of PDAC involve a series of specific genetic alterations which promote growth and survival of aberrant precursors, initiation of a desmoplastic reaction in the stroma, and ultimately tissue invasion and metastases [12]. This oncogenic process begins with transformation of normal pancreatic duct epithelium into infiltrating cancer through a series of histologically defined precursors called pancreatic intraepithelial neoplasia (PanIN)-1, -2, and -3 [11]. These morphological changes occur in conjunction with several genetic alterations. Disease progression often involves development of distant metastases, which occurs late during the genetic evolution of pancreatic cancer [13]. Using genome sequencing, it has been determined that after the initiation of tumorigenesis, an average of 11.7 years is required for the birth of parental, non-metastatic founder pancreatic cancer clone, additional 6.8 years for the development of cancer cell subclones with metastatic potential, and an average of 2.7 years from then until the patient’s death [13].

It is now well established that pancreatic cancer cells contain one or more of the four primary genetic mutations that drive pancreatic cancer tumorigenesis [14]. These include *KRAS*, p16/*CDKN2A*, *TP53*, and *SMAD4* mutations. *KRAS* plays a critical role in regulating important cellular functions including cell survival, cell differentiation, and proliferation [15]. Single point mutations in codon 12, 13, 59, or 61 of exon 2 and exon 3 of the *KRAS* oncogene lead to uncontrolled downstream signaling of RAF/MEK/ERK, leading to enhanced tumor cell proliferation and survival. It has been shown that these activating mutations in the *KRAS* are a necessary event for the initiation of pancreatic cancer and are therefore commonly found in the early precursor lesions (PanIN-1) [16,17]. With disease progression, the prevalence of oncogenic *KRAS* mutation increases and is present in over 90% of the tumors [17,18]. Inactivating mutation in the *CDKN2A* tumor suppressor gene results in the loss of p16 protein and thereby loss of regulation of the G1/S transition of the cell cycle. It is also thought to be a relatively early event in PDAC progression (PanIN-2 lesions) and is associated with larger tumors and early metastasis [17,19]. *TP53* is a DNA checkpoint regulator in response to mutations from reactive oxygen species as well as telomere shortening. Abnormal *TP53* gene allows cells to avoid DNA damage control checkpoints and subsequently apoptotic signals [20]. *SMAD4* is a key component of the transforming growth factor-β (TGF-β) receptor signaling pathway and plays a role in activating transcription of cell cycle inhibitory factors. Inactivation of *TP53* and *SMAD4* occur at a later stage (PanIN-3) in pancreatic carcinogenesis [17].

A comprehensive genome analysis of 24 different human pancreatic cancers revealed an average of 63 genetic alterations per cancer, the majority of which were point mutations [2]. These mutations occur in several primary oncogenes and tumor suppressor genes and contribute to the genetic diversity of pancreatic cancer. This, in turn, leads to tumor heterogeneity, instability, and early metastasis. The genetic alterations associated with pancreatic cancer can be classified into a set of 12 core cellular signaling pathways: apoptosis, control of G1/S phase transition, sonic hedgehog (SHH) signaling, KRAS signaling, TGF-β signaling, Wnt/Notch signaling, DNA damage control, homophilic cell adhesion, integrin signaling, JNK signaling, invasion, and small GTPase signaling [2]. These pathways are responsible for some of the key cellular functions such as intracellular signaling, cell cycle regulation, metabolism, and DNA repair. Targeting these pathways has now become the main focus of drug development in pancreatic cancer.

A prominent histologic hallmark of PDAC is the presence of a desmoplastic reaction which consists of extracellular proliferation of leukocytes, fibroblasts, endothelial cells, neuronal cells, and collagen. This is mediated by paracrine signals from the pancreatic cancer cells which results in the formation of a dense stroma in the tumor microenvironment [21]. It is now established that the signals that promote this stromal reaction originate from the *KRAS*-mutant oncogene in the epithelium of pancreatic cancer cells. SHH signaling also acts in a paracrine fashion on the extracellular fibroblasts, resulting in their growth and differentiation [22]. The desmoplastic reaction not only acts as a mechanical barrier to the effective delivery of chemotherapeutic agents, it also provides an antiangiogenic and hypoxic microenvironment in which the pancreatic cancer cells like to grow and flourish.

Thus, it is now established beyond doubt that the wide range of genetic alterations and the stromal reaction play an important role in the initiation, progression, chemotherapeutic resistance, and recurrence of pancreatic cancer.

### Clinical management

#### Localized disease

The recommended treatment for patients presenting with localized disease is surgery, since complete surgical resection with negative margins offers the only hope for cure in pancreatic cancer treatment [23]. Unfortunately, only 9% of the pancreatic cancer patients present with localized disease that is completely resectable at the time of initial diagnosis [1]. Depending upon the size and the location of the tumor, the operative procedure is either a cephalic pancreaticoduodenectomy (Whipple procedure), distal pancreatectomy, or total pancreatectomy [24]. The success of surgical resection depends on factors such as extent of lymph node involvement, tumor grade, tumor size, CA 19-9 levels, and the positivity of resection margins. Even following complete surgical resection, the 5-year survival rates are low at approximately 20%, and the overall prognosis remains discouraging [23]. Thus, postoperative treatment in the form of adjuvant chemotherapy [25] or chemoradiotherapy [26,27] is usually administered and is often gemcitabine- or 5-fluorouracil (5-FU)-based. The Charité Onkologie Clinical Studies in Gastrointestinal Cancers-001 (CONKO-001) trial randomized 354 patients with localized PDAC to receive either adjuvant gemcitabine or undergo observation after curative resection [28]. Gemcitabine arm was associated with a significant improvement in disease-free survival (DFS; 13.4 months vs. 6.9 months; *P* < 0.001). The median overall survival (OS) was however similar in the two arms (22.1 months vs. 20.2 months; *P* = 0.06) and was explained by the administration of gemcitabine to patients in the observation arm after disease progression. The European Study Group for Pancreatic Cancer (ESPAC)-3 trial compared gemcitabine vs. 5-FU in the adjuvant treatment of PDAC [25]. In this trial, 1,088 patients were randomized to receive either 5-FU/Leucovorin (LV) or gemcitabine. There was no difference in the median OS (23 months vs. 23.6 months; *P* = 0.39), progression-free survival (PFS; 14.1 months vs. 14.3 months; *P* = 0.53), and the global quality of life (QoL) scores between the treatment groups. In the Radiation Therapy Oncology Group (RTOG)-9704 trial, 451 patients with resected PDAC received either gemcitabine or 5-FU chemotherapy before and after 5-FU-based chemoradiation [29]. Among patients with pancreatic head tumors, a statistically non-significant improvement in median OS was seen in the gemcitabine containing arm (20.5 months vs. 16.9 months; *P* = 0.09). A phase III adjuvant trial (UNICANCER) comparing gemcitabine vs. modified FOLFIRINOX in surgically resected (R0 or R1) PDAC patients is currently ongoing (NCT01526135). Another phase III RTOG-0848 trial is comparing adjuvant gemcitabine with or without erlotinib in the first randomization and additional benefit of chemoradiation in the second randomization for patients with localized PDAC who have undergone R0 or R1 surgical resection (NCT01013649).

Another potential strategy for the treatment of localized pancreatic cancer is to administer chemotherapy in the neoadjuvant setting, and this approach has also been explored in multiple phase II clinical trials [30-34]. The rationale for this approach includes early treatment of micrometastatic disease, delivery of chemotherapy to an undisturbed tumor, and biological assessment of tumor aggressiveness [35]. An ongoing phase III study is comparing neoadjuvant gemcitabine/oxaliplatin combination vs. upfront surgery, to be followed by adjuvant gemcitabine in patients with resectable PDAC (NCT01314027).

#### Locally advanced pancreatic cancer

Locally advanced pancreatic cancer (LAPC) is the tumor that is confined locoregionally with some degree of involvement of the nearby major vascular structures but without any evidence of distant metastases. Approximately 30% of the patients are found to have locally advanced stage at the time of initial pancreatic cancer diagnosis [1]. Patients are usually classified as having either borderline-resectable or unresectable LAPC, depending upon the relationship of the tumor with the nearby vascular structures. Unfortunately, there are no standard guidelines for the management of LAPC. This is mainly due to the absence of a single standardized definition of LPAC, which limits a fair comparison of treatment strategies and results across the clinical trials.

The only potential way to achieve a cure in patients with LAPC is by maximizing upfront systemic and local therapy followed by a R0 surgical resection. For borderline-resectable LAPC patients that are deemed ineligible for upfront surgery, neoadjuvant treatment in the form of chemotherapy with or without radiation is usually performed [36-39]. Such neoadjuvant therapies have the potential to downstage borderline-resectable disease and make R0 resection feasible. For initially unresectable LAPC, neoadjuvant therapy should be offered as a bridge to potentially curative resection. Neoadjuvant therapy options include concurrent chemoradiation, chemotherapy alone, and chemotherapy followed by chemoradiation. A 1981 trial conducted by the Gastrointestinal Tumor Study Group (GITSG) established the superiority of combined 5-FU/radiation when compared with radiation therapy alone in unresectable LAPC [40]. Subsequently, gemcitabine-based chemoradiation was evaluated and established as an acceptable treatment option for LAPC, based on the results of the Eastern Cooperative Oncology Group (ECOG)-4201 trial [41] and the Taipei trial [42]. Chemotherapy alone is another management strategy in the neoadjuvant treatment of LAPC. Combination chemotherapy regimens consisting of gemcitabine backbone have failed to demonstrate survival advantage over single agent gemcitabine [43-45]. Non-gemcitabine-based regimens such as FOLFIRINOX and FOLFOX have also been explored in the neoadjuvant treatment of LAPC [46-50]. Yet another approach in the management of LAPC is chemotherapy followed by chemoradiotherapy prior to surgery. Such strategy has been looked at in retrospective series with encouraging results, but prospective phase II/III studies are needed before it can be incorporated into standard oncologic practice [50-52]. The four-arm phase III CONKO-7 trial will evaluate the efficacy of neoadjuvant chemotherapy plus chemoradiation vs. chemotherapy alone in an estimated 830 unresectable LAPC patients (NCT01827553). The chemotherapy options in this trial include either FOLFIRINOX or gemcitabine. A similar phase III trial in LAPC patients to evaluate neoadjuvant modified FOLFIRINOX followed by chemoradiation (with 5-FU as the radiosensitizer) is planned by the investigators from the Stanford University (NCT01926197).

#### Metastatic disease

Approximately 50%–60% of PDAC patients present with metastatic disease at the time of initial diagnosis [1], and the standard treatment option for these patients is chemotherapy. In 1997, gemcitabine monotherapy was established as the standard of care for metastatic disease. In this pivotal trial, single agent gemcitabine (1,000 mg/m^2^ weekly × 7 followed by 1 week of rest, then weekly × 3 every 4 weeks thereafter; *n* = 63) was compared with weekly bolus 5-FU (600 mg/m^2^; *n* = 63), and a modest survival benefit was demonstrated in the gemcitabine group (5.6 vs. 4.4 months; *P* = 0.0025) [53]. The primary efficacy measure was clinical benefit response (CBR), which was a composite of measurements of pain, Karnofsky Performance Status (KPS), and weight. The CBR was experienced by 23.8% of the gemcitabine-treated patients compared with 4.8% of 5-FU-treated patients (*P* = 0.0022). Despite the marginal improvement in 1-year survival, gemcitabine replaced 5-FU and was approved by the US Food and Drug Administration (FDA) as a standard treatment option for the treatment of metastatic pancreatic cancer largely based upon its efficacy in improving the disease-related symptoms [53]. Thereafter, gemcitabine continued as the chemotherapeutic standard of care in the management of patients with metastatic pancreatic cancer for a long time.

Erlotinib, an epidermal growth factor receptor (EGFR) tyrosine kinase inhibitor (TKI), is the only biologic agent that has been approved for the treatment of advanced pancreatic cancer (APC). In the phase III PA.3 trial that randomized 569 patients with locally advanced and metastatic PDAC, the addition of erlotinib to gemcitabine resulted in a statistically significant improvement in median OS of approximately 10 days (6.24 months vs. 5.91 months; hazard ratio [HR] 0.82; *P* = 0.038) [54]. Despite the clinically insignificant benefit, the combination of gemcitabine plus erlotinib received the FDA approval in November 2005 due to a lack of more effective treatment options for this devastating disease at the time. However, due to the associated side effects and an exceedingly small clinical benefit, the use of this regimen in routine oncologic practice has remained virtually non-existent.

In recent years, the evidence has shifted from using single agent gemcitabine to combination regimens for front-line treatment of metastatic pancreatic cancer. FOLFIRINOX and gemcitabine plus nab-paclitaxel have now been established as the two standard combination chemotherapy options for metastatic disease. In the randomized phase III PRODIGE 4/ACCORD 11 trial that consisted of 342 metastatic PDAC patients, FOLFIRINOX (oxaliplatin 85 mg/m^2^, irinotecan 180 mg/m^2^, LV 400 mg/m^2^, and 5-FU 400 mg/m^2^ given as a bolus followed by 2,400 mg/m^2^ given as a 46-hour continuous infusion every 2 weeks) when compared with gemcitabine alone (1,000 mg/m^2^ weekly for 7 of 8 weeks and then weekly for 3 of 4 weeks) demonstrated a significantly better median OS (11.1 vs. 6.8 months; HR 0.57; 95% confidence interval [CI] 0.45 to 0.73; *P* < 0.001), PFS (6.4 months vs. 3.3 months; HR 0.47; 95% CI 0.37 to 0.59; *P* < 0.001), and objective response rate (ORR; 31.6% vs. 9.4%; *P* < 0.001) [55]. Based on the statistically significant and clinically meaningful improvement in the survival, FOLFIRINOX was approved for the first-line treatment of metastatic PDAC. This improvement in OS with FOLFIRINOX is, however, associated with significant toxicity (febrile neutropenia, thrombocytopenia, peripheral neuropathy, vomiting, and diarrhea), and therefore, this regimen is only indicated for patients with good performance status. To improve the tolerability of FOLFIRINOX, a modified regimen was recently proposed in which the 5-FU bolus is removed and growth factor prophylaxis is administered routinely [56].

An important contributor to the chemoresistance of PDAC is the presence of a dense stroma in the tumor microenvironment. Nab-paclitaxel is albumin-bound paclitaxel that has been developed to diminish the stromal tissue network. The ‘albumin’ in nab-paclitaxel interacts with secreted protein acidic and rich in cysteine (SPARC), a matrix glycoprotein that has a role in tumor invasion and facilitates the uptake of paclitaxel by the tumor cells [57]. In a phase I/II trial of 67 patients with metastatic PDAC, nab-paclitaxel plus gemcitabine combination was associated with an ORR of 48%, median OS of 12.2 months, and 1-year survival of 48% [58]. Subsequently, in a large phase III MPACT trial, 861 metastatic PDAC patients were randomized to receive nab-paclitaxel (125 mg/m^2^) followed by gemcitabine (1,000 mg/m^2^) on days 1, 8, and 15 every 4 weeks, vs. gemcitabine monotherapy (1,000 mg/m^2^) weekly for 7 of 8 weeks (cycle 1) and then on days 1, 8, and 15 every 4 weeks (cycle 2 onwards) [59]. The combination of nab-paclitaxel plus gemcitabine significantly improved the median OS from 6.7 months to 8.5 months (HR 0.72; 95% CI 0.62 to 0.83; *P* < 0.001), PFS from 3.7 months to 5.5 months (HR 0.69; 95% CI 0.58 to 0.82; *P* < 0.001) and RR from 7% to 23% (*P* < 0.001) when compared with gemcitabine alone. In September 2013, the FDA approved nab-paclitaxel plus gemcitabine as a first-line treatment option for metastatic pancreatic cancer based on the results of this study. Currently, the choice between the two approved standard first-line chemotherapy options for metastatic disease (FOLFIRINOX or gemcitabine/nab-paclitaxel) is usually guided by the patient’s age, performance status, preference of treatment frequency, and toxicity profiles of the two regimens.

### Emerging novel therapeutic targets and treatment strategies

Currently, a multitude of innovative therapeutic approaches are being developed to target the known molecular pathways involved in pancreatic tumorigenesis. Table 1 provides a list of the selected clinical trials that are currently recruiting patients to evaluate the safety and efficacy of novel agents in PDAC.

**Table 1:** **Summary of selected novel agents that are under evaluation in currently actively recruiting clinical trials**

**Primary modes of action**	**Study agent(s)**	**NCT identifier**	**Phase**	**Disease stage**
MEK inhibitor + Bcl-2 inhibitor	Trametinib, navitoclax	NCT02079740	Ib/II	LA, metastatic
MEK inhibitor + ErbB inhibitor	PD-0325901, dacomitinib	NCT02039336	I/II	Metastatic
	Trametinib, lapatinib	NCT02230553	I/II	Metastatic
EGFR TKI	Afatinib	NCT01728818	II	Metastatic
PI3K inhibitor	BKM120	NCT01571024	I	Metastatic
	BYL719	NCT02155088	I	LA, metastatic
PI3K inhibitor + MEK inhibitor	BYL719, MEK162	NCT01449058	Ib/II	Metastatic
AKT inhibitor	MK2206	NCT01783171	I	LA, metastatic
PTEN inducer	AXP107-11	NCT01182246	Ib/II	LA, metastatic
Wnt signaling inhibitor	OMP-54 F28	NCT02050178	Ib	Metastatic
	OMP-18R5	NCT02005315	Ib	Metastatic
	PRI-724	NCT01764477	I	LA, metastatic
	LGK974	NCT01351103	I	LA, metastatic
Glycogen synthase kinase-3 inhibitor	LY2090314	NCT01632306	I, II	Metastatic
Notch signaling inhibitor	MK0752	NCT01098344	I	LA, metastatic
	PF-03084014	NCT02109445	Ib/II	Metastatic
	OMP-59R5	NCT01647828	Ib/II	Metastatic
	OMP-21 M18	NCT01189929	Ib	LA, metastatic
Anti-connective tissue growth factor mAb	FG-3019	NCT02210559	II	LA unresectable
Heparan sulfate mimetic	M402	NCT01621243	I/II	Metastatic
Hyaluronidase	PEGPH20	NCT01839487	II	Metastatic
		NCT01959139	I/II	Metastatic
Hyaluronidase + anti-EGFR mAb	PEGPH20, cetuximab	NCT02241187	NP	Resectable
Oncolytic adenovirus encoding hyaluronidase	VCN-01	NCT02045589	I	LA, metastatic
Hedgehog inhibitor	IPI-926	NCT01383538	I	LA, metastatic
	GDC-0449	NCT01088815	II	Metastatic
	LDE-225	NCT01431794	I/II	LA
Hypoxia targeting agent	TH-302	NCT02047500	I	LA, metastatic
TGF-β receptor I inhibitor	LY2157299	NCT01373164	Ib/II	LA, metastatic
Hypomethylating agent	Azacitidine	NCT01845805	II	Resected
AMP-activated protein kinase (AMPK) activator	Metformin	NCT01954732	I	Localized
	Metformin	NCT02005419	II	Localized
	Metformin	NCT01666730	II	Metastatic
AMPK activator + mTOR inhibitor	Metformin, Rapamycin	NCT02048384	Ib/II	Metastatic
poly (ADP-ribose) polymerase (PARP) inhibitor	Veliparib	NCT01908478	I	LA
	Veliparib	NCT01489865	I/II	Metastatic
	Veliparib	NCT01585805	II	LA, metastatic
	Rucaparib (AG-14699)	NCT02042378	II	LA, metastatic (BRCA mutant)
	Olaparib (AZD2281)	NCT02184195	III	Metastatic (BRCA mutant)
Vascular targeting agent	ADH-1	NCT01825603	I	LA, metastatic
Antiangiogenic combination	Tl-118	NCT01509911	II	Metastatic
Arginine degrading enzyme	ADI-PEG 20	NCT02101580	Ib	LA, metastatic
Aurora A kinase inhibitor	Alisertib (MLN8237)	NCT01924260, NCT01677559	I	LA, metastatic
CDK inhibitor + AKT inhibitor	Dinaciclib, MK2206	NCT01783171	I	LA, metastatic
α-ketoglutarate dehydrogenase (KGDH) inhibitor	CPI-613	NCT01835041	I	Metastatic
	CPI-613	NCT01839981	I	LA, metastatic
c-Met inhibitor	Cabozantinib (XL184)	NCT01663272	I	LA, metastatic
CRM-1 inhibitor	Selinexor (KPT-330)	NCT02178436	Ib/II	Metastatic
DNA minor groove binder	Lurbinectedin	NCT02210364	I	LA unresectable
Src inhibitor	Dasatinib	NCT01652976	II	Metastatic
Trk A, B, C inhibitors	PLX7486	NCT01804530	I	LA, metastatic
IDO inhibitor	Indoximod	NCT02077881	I/II	Metastatic
	NLG919	NCT02048709	I	Refractory
Chemokine receptor 2 (CCR2) antagonist	PF-04136309	NCT01413022	I	LA
Anti-tissue factor mAb	MORAb-066	NCT01761240	I	LA, metastatic
Wee 1 inhibitor	MK1775	NCT02037230	I/II	LA unresectable
BET bromodomain inhibitor	OTX015	NCT02259114	Ib	LA, metastatic
SMAC mimetic	LCL161	NCT01934634	I	Metastatic
Cancer stemness inhibitor	BBI608	NCT02231723	Ib	Metastatic
Janus Kinase (JAK) inhibitor	Ruxolitinib	NCT01822756	I	Metastatic
	Ruxolitinib	NCT02117479, NCT02119663	III	Metastatic
	INCB039110	NCT01858883	Ib	Metastatic
	Momelotinib	NCT02101021	II	Metastatic
Autophagy inhibitor	Hydroxychloroquine	NCT01506973	I/II	Metastatic
	Hydroxychloroquine	NCT01978184	II	Resectable
	Hydroxychloroquine	NCT01494155	II	Resectable
Cancer vaccine	GVAX	NCT01088789	II	Localized
	Poly ICLC and dendritic cells	NCT01677962	0	LA unresectable
	Autologous tumor-derived HSP gp96	NCT02133079	I/II	Resected
	GVAX/CRS-207	NCT02004262	II	Metastatic
	Algenpantucel-L	NCT01836432	III	LA
hTERT DNA cancer vaccine	INO-1400	NCT02327468	I	Non-metastatic
CTLA-4 inhibitor	Ipilimumab	NCT01473940	Ib	LA, metastatic
Anti-PD-1 mAb	CT-011	NCT01313416	II	Resected
Vaccine + CTLA-4 inhibitor	GVAX, ipilimumab	NCT01896869	II	Metastatic
Vaccine + anti-PD-1 mAb	GVAX/CRS-207, nivolumab	NCT02243371	II	Metastatic
CTLA-4 inhibitor + anti-PD-1 mAb	Ipilimumab, nivolumab	NCT01928394	I/II	LA, metastatic
Anti-CPAA mAb	NPC-1C	NCT01834235	I/II	LA, metastatic
	NPC-1C	NCT01040000	II	LA, metastatic
Anti-MUC1 mAb	BTH1704	NCT02132403	I	LA, metastatic
Anti-CEA BiTE mAb	MEDI-565	NCT01284231	I	Refractory
Anti-CA-125 mAb	Oregovomab	NCT01959672	II	Non-metastatic
Pegylated recombinant human IL-10	AM0010	NCT02009449	I	Metastatic
IL-1 receptor antagonist	Anakinra	NCT02021422	I	Metastatic
RAS specific immunotherapy	TG01	NCT02261714	I/II	Resected
Radioimmunotherapy	90Y-clivatuzumab tetraxetan (IMMU-107)	NCT01956812	III	Metastatic
Activated T-cells	EGFRBi armed ATC infusions	NCT01420874	I	Metastatic
Dendritic cell/cytokine-induced killer cells	DC-CIK	NCT01781520	I/II	LA, metastatic
siRNA-transfected PBMC	APN401	NCT02166255	I	LA, metastatic
Autologous CAR T-cells	RNA mesothelin re-directed CAR T-cells	NCT01897415	I	Metastatic
	Anti-mesothelin gene engineered lymphocytes	NCT01583686	I/II	Metastatic
Autologous natural killer T-cells	NKT cells	NCT01801852	I	Refractory
Activated dendritic cells	DCVax-Direct	NCT01882946	I/II	LA, metastatic
Autologous tumor infiltrating lymphocytes + interleukin	TIL, IL-2	NCT01174121	II	Metastatic
Antiguanylyl cyclase C antibody-drug conjugate (ADC)	MLN0264	NCT02202785	II	LA, metastatic
Micellar nanoparticle-encapsulated cisplatin	NC-6004	NCT02043288	III	LA or metastatic
Alkylating agent	Glufosfamide	NCT01954992	III	Metastatic

Source: http://www.clinicaltrials.gov; Accessed on January 01, 2015. *NCT* National Clinical Trial, *EGFR* epidermal growth factor receptor, *TKI* tyrosine kinase inhibitor, *PTEN* Phosphatase and tensin homolog, *TGF* transforming growth factor receptor, *mAb* monoclonal antibody, *CRM1* chromosome region maintenance 1, *DNA* deoxyribonucleic acid, *BET* bromodomain and extra-terminal, *SMAC* second mitochondrial-derived activator of caspases, *hTERT* telomerase reverse transcriptase, *CTLA-4* cytotoxic T-lymphocyte-associated protein 4, *PBMC* peripheral blood mononuclear cell, *siRNA* small interfering RNA, *LA* locally advanced.

#### RAS/RAF/MEK/ERK signaling pathway

Despite being the most common mutation associated with PDAC, attempts to target *KRAS* by inhibiting its post-translational modification have been unsuccessful so far. Tipifarnib (R115777) is an inhibitor of farnesyltransferase (FTase) which is a dominant enzyme involved in post-translational modification of RAS [60]. So far, it has not demonstrated any significant antitumor activity both as a single agent and in combination with gemcitabine [44,61,62].

Attempts are being made to identify downstream targets (mitogen-activated protein kinase [MAPK], phosphatidylinositide 3-kinase [PI3K]) to block KRAS-dependent signaling pathways. Towards this goal, a number of MEK inhibitors are currently being evaluated in clinical trials [63]. Selumetenib (AZD6244) is a selective MEK inhibitor that was found to have similar efficacy as capecitabine in a phase II clinical trial that enrolled APC patients after failing first-line gemcitabine therapy [64]. It is also being tested in combination with erlotinib in APC patients resistant to gemcitabine (NCT01222689). Based on the encouraging results of a phase I clinical trial, trametinib (another MEK inhibitor) was combined with gemcitabine in a phase II clinical trial of metastatic pancreatic cancer patients but failed to demonstrate a clinical benefit [65]. Combinations of other novel MEK inhibitors (pimasertib [MSC1936369B], refametenib [BAY86-9766]) with gemcitabine are currently under evaluation in clinical trials.

It is now known that expression of RAS oncogene up-regulates basal autophagy, which is required for cancer cell survival in starvation and in tumorigenesis [66]. Autophagy is therefore believed to be a significant mechanism for pancreatic cancer cell survival. Hydroxychloroquine, an anti-malarial drug is being evaluated as an autophagy inhibitor for the treatment of these aggressive cancers.

#### Epidermal growth factor receptor pathway

ErbB-1 (EGFR) and ErbB-2 (HER2/neu) expression is found in 90% and 21% of pancreatic cancers, respectively [67,68]. Therapies targeted against EGFR (both TKIs and monoclonal antibody [mAb]) in pancreatic cancer have yielded overall disappointing results so far. As discussed previously, the phase III PA.3 trial evaluated gemcitabine in combination with erlotinib in the first-line treatment of APC and was associated with a clinically insignificant improvement in median OS when compared with gemcitabine alone [54]. In another phase II study of gemcitabine-refractory APC patients, a combination of erlotinib and capecitabine was associated with only 10% radiological response and a median OS of 6.5 months [69]. The combination of cetuximab and gemcitabine has also been evaluated in the treatment of APC patients [70,71]. The initial phase II study demonstrated stable disease (SD) in 63% and partial response (PR) in 12% of the EGFR-expressing APC patients that were treated with cetuximab plus gemcitabine combination [70]. In a subsequent phase III study (Southwest Oncology Group [SWOG]-directed intergroup trial S0205), this combination was not associated with any survival benefit when compared with the single agent gemcitabine (6.3 months vs. 5.9 months; HR 1.06; 95% CI 0.91 to 1.23; *P* = 0.23) [71]. A randomized phase II study of panitumumab, erlotinib, and gemcitabine combination suggested a trend towards OS benefit when compared with erlotinib plus gemcitabine [72]. However, this three-drug combination with dual inhibition of the EGFR pathway was associated with significant toxicities leading to early termination of the study. Anti-HER2 agent trastuzumab has been combined with gemcitabine in a phase II study that included metastatic pancreatic cancer patients with 2+ (88% patients) or 3+ (12% patients) HER2/neu overexpression by immunohistochemistry [73]. The response rate of this combination was very similar to gemcitabine alone.

One of the probable explanations for the lack of a meaningful benefit from anti-EGFR TKIs in pancreatic cancer could be the development of acquired resistance to these agents, which is a mechanism well studied in lung cancer [74]. Clinical trials evaluating newer EGFR TKIs such as afatinib (NCT01728818) and dacomitinib (NCT02039336) in pancreatic cancer are currently underway.

#### Anti-angiogenesis

Targeting vascular endothelial growth factor (VEGF) pathway has shown promising results in the treatment of many solid cancers. However, anti-VEGF therapies have been ineffective clinically in treating patients with PDAC. The phase III Cancer and Leukemia Group B (CALGB) 80303 trial randomized 602 patients with APC to receive gemcitabine with or without bevacizumab in the first-line setting [75]. The addition of bevacizumab to gemcitabine was associated with increased toxicity and without any improvement in survival (5.8 months vs. 5.9 months; *P* = 0.95). In another large, randomized phase III trial (AVITA), 607 metastatic pancreatic cancer patients were randomized to receive gemcitabine plus erlotinib with either bevacizumab or placebo [76]. The bevacizumab arm was associated with statistically significant PFS advantage (4.6 months vs. 3.6 months; HR 0.73; *P* = 0.0002) but a non-significant improvement in median OS (7.1 months vs. 6 months; HR 0.89; *P* = 0.2087). Axitinib is a selective oral inhibitor of VEGF receptor-1, -2, and -3 that has been combined with gemcitabine in a phase II clinical trial of APC patients and showed a statistically non-significant gain in OS [77]. A subsequent phase III study that randomized 632 APC patients to receive gemcitabine plus axitinib or placebo was terminated early due to the lack of survival benefit (8.5 months vs. 8.3 months; HR 1.014; *P* = 0.5436) at the time of planned interim analysis [78]. Ziv-Aflibercept is an anti-VEGF recombinant fusion protein that has also been combined with gemcitabine in a phase III trial for the treatment of metastatic pancreatic cancer patients [79]. However, this trial was terminated early as well due to the lack of efficacy at the time of planned interim analysis. Sorafenib and masitinib are oral multikinase inhibitors with antiangiogenic properties. In the phase III BAYPAN trial, addition of sorafenib to gemcitabine did not improve PFS in APC patients [80]. The phase III study of gemcitabine plus masitinib also did not result in improvement of OS in patients with unresectable pancreatic cancer [81].

#### Insulin-like growth factor-1 receptor

Insulin-like growth factor (IGF)-1 receptor is highly expressed in PDAC and participates in downstream signaling pathways that are involved in cancer cell survival and proliferation. Several mAbs against IGF-1 receptor (cixutumumab, ganitumab, dalotuzumab) are currently being tested in clinical trials. Ganitumab (AMG 479) was studied in combination with gemcitabine in a phase II trial of metastatic pancreatic cancer patients and showed a slight improvement in 6-month survival rate when compared with gemcitabine plus placebo (57% vs. 50%) [82]. However, the phase III GAMMA trial of ganitumab plus gemcitabine combination was terminated early due to lack of efficacy at the preplanned interim analysis. In another phase II trial that evaluated cixutumumab plus gemcitabine and erlotinib in metastatic pancreatic cancer patients, the three-drug combination did not improve the PFS or OS when compared with gemcitabine plus erlotinib [83].

#### PI3K/AKT/mTOR pathway

This is one of the major downstream effector pathways of *KRAS* gene that is being evaluated as a potential target for pancreatic cancer treatment [84]. Rigosertib is a small molecular inhibitor of PI3K that was combined with gemcitabine in a phase II/III clinical trial (ONTRAC trial). The study was terminated early due to lack of demonstration of benefit at the time of interim analysis. Buparlisib (BKM120) is another PI3K inhibitor being evaluated in combination with mFOLFOX6 regimen in a study of advanced stage solid tumors including pancreatic cancer (NCT01571024). MK2206 is an AKT inhibitor currently under clinical evaluation in patients with pancreatic cancer (NCT01783171, NCT01658943). Archexin (RX-0201) is another AKT inhibitor that was evaluated in combination with gemcitabine in a phase II study (NCT01028495). BEZ235 is a combined inhibitor of PI3K and mTOR. A phase I study evaluating the activity of BEZ235 plus a MEK inhibitor (MEK162) in advanced solid tumor patients (including pancreatic cancer) with *KRAS*, *NRAS* and/or *BRAF* mutations has recently completed (NCT01337765). Everolimus (RAD001) is a mTOR inhibitor that was associated with a PFS of 1.8 months and OS of 4.5 months in a phase II study consisting of 33 gemcitabine-refractory, metastatic pancreatic cancer patients [85]. It is also being evaluated as a part of combination regimens with other agents in ongoing clinical trials.

#### Wnt/β-catenin pathway

Wnt signals are transduced through the frizzled receptor and lipoprotein-related protein to the β-catenin signaling cascade. There is evidence to suggest that Wnt pathway plays a role in pancreatic cancer formation via involvement in pancreatic cancer stem cells (CSCs) [86,87]. Phase I trials using mAbs (OMP-54 F28, OMP-18R5) against frizzled receptors to inhibit Wnt signaling in PDAC are currently ongoing (NCT02050178, NCT02005315).

#### Notch signaling pathway

Notch signaling has been shown to be upregulated in many human cancers including PDACs [88,89]. It mediates pancreatic CSC function and contributes to chemotherapy resistance, tumor recurrence, and metastases. Gamma secretase is an enzyme that causes proteolytic cleavage and release of the intracellular domain of the Notch, leading to activation of the Notch signaling pathway. In preclinical models, inhibition of Notch pathways with a gamma-secretase inhibitor (GSI) in combination with gemcitabine showed enhanced antitumor activity [90]. A phase II study evaluating an oral GSI (RO4929097) in pretreated metastatic pancreatic cancer patients was recently completed (NCT01232829). MK0752 is another GSI being tested in combination with gemcitabine for first-line treatment of stage III and IV PDAC patients (NCT01098344). Tarextumab (anti-Notch2/3 mAb, OMP-59R5) and demcizumab (anti-DLL4 mAb, OMP-21 M18) also inhibit Notch signaling and are being evaluated in clinical trials. ALPINE trial is studying the combination of tarextumab with nab-paclitaxel and gemcitabine (NCT01647828).

#### Targeting desmoplastic tumor microenvironment

The desmoplastic stroma in the tumor microenvironment is now regarded as a key component of pancreatic cancer biology which not only acts as a physical barrier to effective drug delivery inside the tumor but also facilitates tumor growth and promotes metastases. Strategies aimed at targeting the stromal compartment may enhance the delivery of chemotherapeutic agents to the tumor cells leading to improved efficacy. The most promising targets include the SHH signaling pathway, hyaluronic acid, and SPARC.

SHH pathway is an important signaling system that can activate the characteristic desmoplastic reaction present in the microenvironment of pancreatic tumors [22]. Sustained activation of this pathway enhances tumor growth during pancreatic oncogenesis [91]. Several clinical trials have been initiated to investigate the activity of SHH inhibitors in patients with PDAC. Vismodegib (GDC-0449) is a SHH inhibitor under clinical evaluation in combination with gemcitabine and nab-paclitaxel (NCT01088815). Saridegib (IPI-926) is another agent targeting the SHH pathway that was combined with gemcitabine in a phase II study of APC, but the trial was prematurely terminated since the combination was associated with a shorter survival than gemcitabine alone (NCT01130142). A study combining the SHH inhibitor sonidegib (LDE225) with FOLFIRINOX in untreated APC patients is ongoing (NCT01485744).

Hyaluronan is a glycosaminoglycan present in the extracellular matrix of PDAC, and high levels within the tumor are usually associated with a poor prognosis. Pegylated human recombinant PH20 hyaluronidase (PEGPH20) degrades hyaluronan and has been shown to decrease the hyaluronic acid content in a genetically-engineered PDAC mouse model, allowing for re-expansion of the PDAC blood vessels and enhanced intratumoral delivery of chemotherapeutic agents which leads to decreased tumor growth [92]. Clinically, the combination of PEGPH20 plus gemcitabine has shown promising activity in a phase Ib study of metastatic pancreatic cancer patients [93], and a phase II study of this combination is ongoing (NCT01453153). Another phase Ib/II study of PEGPH20 plus modified FOLFIRINOX combination in metastatic pancreatic cancer patients is currently under clinical evaluation (NCT01959139).

SPARC (osteonectin) is an extracellular matrix protein that plays a role in collagen turnover in the dense stroma. It is associated with invasion and metastasis in PDAC, and elevated levels are associated with poor prognosis. Nab-paclitaxel is albumin-bound paclitaxel that increases tumor accumulation of paclitaxel through binding of albumin to the stroma rich in overexpression of SPARC. As described previously, the efficacy of nab-paclitaxel plus gemcitabine combination in the first-line treatment of metastatic pancreatic cancer was demonstrated in the phase III MPACT trial which ultimately led to its FDA approval [59].

Some of the other novel strategies aimed at targeting the desmoplastic stroma within the pancreatic tumor include the use of matrix metalloproteinase (MMP) inhibitors, heparin derivatives, and hypoxia targeting agents. MMP inhibitors have been tried for the treatment of PDAC without much success to date. Marimastat (BB-2516) is a broad spectrum MMP inhibitor that was combined with gemcitabine in the treatment of APC but did not show any demonstrable clinical benefit [94]. Tanomastat (BAY12-9566) is another biphenyl MMP inhibitor with antiangiogenic and antimetastatic properties that was compared with gemcitabine for the treatment of APC patients and was found to be inferior to gemcitabine [95]. Heparin-derivative agents such as 2-0, 3-0 desulfated heparin (ODSH) and necuparanib (M402) are currently being studied in combination with gemcitabine and nab-paclitaxel for treatment of patients with metastatic pancreatic cancer (NCT01461915, NCT01621243). It is now well established the pancreatic tumor microenvironment is characterized by hypoxia. Consequently, hypoxia-targeting agents are being developed to evaluate this novel therapeutic strategy. TH-302 is a hypoxia-activated prodrug that is activated into a potent DNA-alkylating agent, bromo-isophosphoramide mustard selectively under hypoxic conditions. A recent phase II study of TH-302 plus gemcitabine showed a significant improvement in primary end point of PFS when compared with gemcitabine alone (5.6 months vs. 3.6 months; HR 0.61; 95% CI 0.43 to 0.87; *P* = 0.005) [96]. A phase III trial of this combination is currently in progress (MAESTRO study; NCT01746979).

#### TGF-β signaling pathway

TGF-β participates in stimulating stromal reaction, invasion, metastases, and angiogenesis in PDAC [97]. Examples of novel agents that target TGF-β signaling include trabedersen (AP-12009) and galunisertib (LY2157299). Trabedersen is a specific inhibitor of TGF-β2 that has demonstrated good safety and encouraging survival results in the phase I/II clinical study [98]. Galunisertib is being evaluated in combination with gemcitabine for the treatment of patients with APC in an ongoing phase Ib/II clinical trial (NCT01373164).

#### Epigenetic modification

Epigenetic changes such as histone deacetylation (HDAC) and DNA methylation (cytosine methylation within CG dinucleotides) can result in inactivation of the tumor suppressor genes leading to tumor growth and progression. Vorinostat is a HDAC inhibitor being tested in a phase I/II study of LAPC patients in combination with capecitabine and radiotherapy (NCT00948688). 5-Azacytidine is a cytosine analog that inhibits DNA methyltransferase, and a phase I study of its combination with gemcitabine in APC patients was recently terminated (NCT01167816).

#### Adenosine monophosphate-activated protein kinase pathway

The oral anti-diabetic drug metformin is an activator of adenosine monophosphate-activated protein kinase (AMPK) and disrupts the crosstalk between insulin receptor and G protein-coupled receptors (GPCR) signaling in pancreatic cancer cells, via inhibition of mTOR and suppression of its downstream effectors [99]. In xenograft mice models, metformin has been shown to inhibit pancreatic cancer growth [99]. Clinically, the available data surrounding the benefit of metformin in pancreatic cancer is conflicting and is mostly derived from retrospective studies. In a hospital-based case-control study, metformin was shown to decrease the risk of developing pancreatic cancer among diabetics [100]. In another retrospective analysis, metformin use was associated with an improvement in survival for the PDAC patients with diabetes [101]. In contrast, a more recent study from the UK failed to demonstrate a survival benefit from metformin use in PDAC patients [102]. There are multiple ongoing phase I and phase II trials that are evaluating the efficacy of metformin in PDAC. A phase I study of metformin plus erlotinib and gemcitabine in patients with APC has recently completed accrual (NCT01210911).

#### Synthetic lethality

The DNA double-strand breaks are repaired by a process of homologous recombination that is mediated via BRCA1 and BRCA2 proteins. Mutations in *BRCA* render this repair mechanism dysfunctional and are known to occur in both sporadic and familial cases of pancreatic cancer. Poly (ADP-ribose) polymerase (PARP) is another critical enzyme that mediates repair of DNA single-strand breaks. PARP pathway assumes the major role for DNA repair when BRCA mutation occurs. Consequently, inhibition of the PARP pathway results in ‘synthetic lethality’ via inhibition of DNA repair in BRCA-deficient tumor cells. A phase II study of veliparib alone or in combination with gemcitabine plus cisplatin for locally advanced and metastatic, *BRCA 1–2*, and *PALB2*-mutated pancreatic cancer patients is ongoing (NCT01585805). Other PARP inhibitors that are being evaluated in pancreatic cancer clinical trials include rucaparib (AG-14699; NCT02042378) and olaparib (AZD2281; NCT00515866).

#### Immunotherapy-based approaches

Despite significant efforts, no immunotherapeutic strategy against pancreatic cancer has demonstrated clinical benefit in a randomized phase III trial till date. This has been attributed to the immunologically quiescent microenvironment of pancreatic cancer. Currently, several approaches aimed at stimulating the host immune system against the pancreatic cancer tumor cells are under evaluation.

GI-4000, a form of RAS-specific immunotherapy, is heat-killed recombinant *Saccharomyces cerevisiae* yeast that expresses mutant RAS peptides [103]. A phase II trial of GI-4000 plus adjuvant gemcitabine is ongoing (NCT00300950). Reovirus is a tumor-targeted replication-competent virus with specificity for RAS-activated cells [104]. It is being combined with chemotherapy for the treatment of APC patients in two phase II clinical trials (NCT00998322, NCT01280058).

Developing vaccines against tumor antigens is another potential immunotherapeutic strategy to treat pancreatic cancer. Several antigens have been explored as potential targets for vaccine-based treatment in pancreatic cancer including carcinoembryonic antigen (CEA) [105], MUC1 [106,107], and heat shock proteins (HSP) [108]. Algenpantucel-L immunotherapy is a whole-cell allogeneic pancreatic cancer vaccine composed of two irradiated human pancreatic cell lines that have been genetically modified to overexpress murine alpha(1,3)-galactosyltransferase, resulting in expression of alpha-galactosyl (Alpha-Gal) epitopes on membrane glycoproteins and glycolipids. Since human cells do not express these epitopes, an immediate hyperacute rejection response ensues leading to the development of strong T-cell mediated antitumor immunity. This immunotherapeutic agent has demonstrated encouraging activity when combined with radiation and 5-FU plus gemcitabine in a phase II adjuvant trial of resected PDAC patients [109]. The phase III adjuvant trial that compares gemcitabine with or without algenpantucel-L, followed by chemoradiation has also completed recently (NCT01072981). Another phase III neoadjuvant trial is evaluating FOLFIRINOX with or without algenpantucel-L, followed by chemoradiation in borderline-resectable and unresectable LAPC patients (NCT01836432). GV1001 is a telomerase peptide vaccine shown to prolong survival when combined with granulocyte-macrophage colony-stimulating factor (GM-CSF) in a phase I/II study of unresectable LAPC patients [110]. However, the phase III study comparing GV1001 and gemcitabine in sequential combination, vs. gemcitabine monotherapy in advanced unresectable pancreatic cancer was terminated early due to lack of survival benefit in the GV1001 arm (NCT00358566). G17D (Gastrimmune) is an antigastrin-17 immunogen that was evaluated in a randomized, multicenter, placebo-controlled study of APC patients and showed a non-significant improvement in OS when compared to placebo [111]. Another potential therapeutic strategy that has been explored is combining two vaccines (GVAX plus CRS-207) with the hope to achieve an enhanced efficacy. GVAX is composed of pancreatic cancer cells that have been genetically modified to secrete GM-CSF and can induce T-cell responses. CRS-207 is a live-attenuated Listeria-based vaccine that can induce listeriolysin O and mesothelin-specific T-cell responses. The combination of GVAX plus CRS-207 is being evaluated in a phase IIb clinical trial (ECLIPSE) that consists of previously treated metastatic pancreatic cancer patients (NCT02004262).

Prostate stem cell antigen (PSCA) is a glycosylphosphatidylinositol-linked cell surface antigen expressed in pancreatic cancers. AGS-1C4D4 is a fully human IgG1 mAb against PSCA that has been combined with gemcitabine for the treatment of metastatic pancreatic cancer patients and has shown encouraging results [112].

It is now established that CD40 activation can reverse immune suppression and drive antitumor T-cell responses. Utilizing this concept, agonist CD40 antibody has been used in combination with gemcitabine in a phase I study to shrink PDAC by stimulating tumor macrophages against pancreatic cancer stroma [113].

Immunoinhibitory checkpoint pathways (cytotoxic T lymphocyte-associated protein-4 [CTLA-4]/B7, programmed cell death-1 [PD-1]/programmed cell death ligand-1 [PD-L1]) are emerging as interesting immunotherapeutic targets for the treatment of cancer. Single agent ipilimumab was evaluated in a phase II trial of APC and failed to demonstrate an appreciable antitumor activity [114]. The combination of ipilimumab with gemcitabine is currently under phase I evaluation (NCT01473940).

Radioimmunotherapy with ^90^Y-clivatuzumab tetraxetan (radioimmunoconjugate comprised of the humanized mAb HuPAM4 that is radiolabeled with yttrium-90) is another potential therapeutic strategy that is being evaluated in clinical trials. The combination of ^90^Y-clivatuzumab tetraxetan with low-dose gemcitabine demonstrated a median OS of 7.7 months in a phase I study of untreated APC patients [115]. The phase III study (PANCRIT-1) of this combination in pretreated metastatic pancreatic cancer patients is ongoing (NCT01956812).

An additional therapeutic strategy is adoptive cell transfer (ACT) approach which utilizes introduction of engineered T-cells with chimeric antigen receptors (CARs) to specifically recognize a tumor antigen of interest. This personalized immunotherapy approach is still under preclinical stages of development in the field of pancreatic cancer [116].

#### Novel cytotoxic agents

PEP02 (MM-398) is a novel nanoparticle liposomal formulation of irinotecan. In a phase II study of gemcitabine-refractory metastatic PDAC patients, treatment with single agent PEP02 was associated with a median PFS and OS of 9 weeks and 21.6 weeks, respectively [117]. A phase III trial (NAPOLI 1) is evaluating the combination of PEP02 with 5-FU in metastatic pancreatic cancer patients who have failed prior gemcitabine-based therapy (NCT01494506).

S-1 is a fourth-generation oral fluoropyrimidine that contains tegafur (FT, a prodrug of 5-FU), 5-chloro-2,4-dihydropyrimidine (CHDP), and potassium oxonate (Oxo). It has been evaluated in the treatment of both resectable and advanced pancreatic cancer with encouraging results. In a phase II Japanese study (PC-01), 116 patients with unresectable APC were randomized to receive gemcitabine plus S-1 vs. gemcitabine alone [118]. There was significant improvement in the ORR (28.3% vs. 6.8%; *P* = 0.005) and median OS (13.7 months vs. 8.0 months; *P* = 0.035) in the S-1 arm. Japan Adjuvant Study Group of Pancreatic Cancer (JASPAC-01) is a phase III non-inferiority trial that compared S-1 with gemcitabine as adjuvant chemotherapy for patients with curatively resected pancreatic cancer [119]. The interim analysis showed that S-1 was non-inferior to gemcitabine (OS at 2 years was 70% vs. 53%; HR 0.56; 95% CI 0.42 to 0.74; *P* < 0.0001 for non-inferiority) [120]. Based on the results of this study, the authors proposed that S-1 should be considered as a new standard treatment for patient with resected pancreatic cancer.

### Identification of biomarkers

Development of more efficacious approaches for pancreatic cancer treatment would require identification of biomarkers that can predict the response and toxicity to various therapeutic agents. Research efforts geared towards this objective are underway.

The human equilibrative nucleoside transporter-1 (hENT1) plays an important role in the uptake of gemcitabine in cells and has been evaluated as a potential predictive biomarker of gemcitabine response. In a study that evaluated the expression pattern of genes involved in gemcitabine activity in 102 pancreatic tumor specimens, it was found that low hENT-1 expression levels were associated with a poorer prognosis [121]. However, in the pivotal phase II Low hENT1 and Adenocarcinoma of the Pancreas (LEAP) study, the hENT1 status was shown to have no clinical utility for predicting gemcitabine sensitivity [122]. Some additional potential biomarkers that have been evaluated in pancreatic cancer treatment include deoxycytidine kinase (dCK), ribonucleoside reductase-M1 (RRM1), and -M2 (RRM2) [123,124], *KRAS* status, SPARC staining [58], IGF-1R expression, and rs9582036 single nucleotide polymorphism (SNP) in the VEGF receptor-1 region [125]. Recently, pharmacogenomic profiling of circulating tumor and invasive cells (CTICs) isolated from patients with PDAC was evaluated as a predictor of tumor response, progression, and resistance [126].

## Future directions and conclusion

The therapeutic advances in the field of pancreatic cancer have been painstakingly slow. Although we have seen a few breakthroughs in therapy for advanced disease in the recent years, the overall progress made in the field of pancreatic cancer has been relatively small in comparison to the some other tumor types. It should be noted that the majority of pancreatic cancer clinical trials over the past 5 years have failed to demonstrate any significant clinical benefit. A substantial fraction of these studies evaluated drug combinations using gemcitabine as the chemotherapy backbone. This should make us think that maybe it is time to move away from gemcitabine-based combinations and focus attention on developing innovative strategies to attack the pancreatic cancer oncogenesis. Selecting drug combinations with novel agents that target not only the primary tumor but also the surrounding stroma might be one such approach. Since our ability to safely combine drugs will be enhanced if the drug selection is based on biomarkers, we would also need prospective studies to validate potential biomarkers in well-defined patient populations in order to maximize the clinical efficacy while minimizing the toxicity of the therapeutic agents.

It is now well established that pancreatic cancer is a heterogeneous and a genetically diverse disease that results from successive accumulation of mutations over a long period of time, and these mutations affect multiple molecular pathways involved in pancreatic tumorigenesis. The presence of desmoplastic reaction in the tumor microenvironment and its role in pancreatic cancer initiation, invasion, and metastases is also being recognized. Consequently, multiple potential therapeutic approaches against pancreatic cancer are being developed and evaluated in several ongoing preclinical and clinical trials. We are hopeful that at least some of these novel strategies will demonstrate clinically meaningful benefit in future phase III studies and add to our armamentarium for treating this lethal malignancy.
